# GWAS-by-subtraction reveals new genetic architecture and health implications of type 2 diabetes-independent gestational diabetes mellitus

**DOI:** 10.1186/s13073-026-01674-2

**Published:** 2026-05-22

**Authors:** Siquan Zhou, Zhichang Ran, Yanliu Li, Jiayuan He, Xin Yuan, Yuanqi Hu, Xiaoyu Wang, Ruirui Li, Yujie Xu, Ciling Yan, Jingyuan Xiong, Guo Cheng

**Affiliations:** 1 West China School of Public Health and West China Fourth Hospital, Sichuan University, 16 Renminnan Road 3rd Section, Chengdu, 610041 China; 2 Laboratory of Molecular Translational Medicine, Center for Translational Medicine, Key Laboratory of Birth Defects and Related Diseases of Women and Children (Sichuan University), Maternal & Child Nutrition Center, West China Second University Hospital, Ministry of Education, Sichuan University, 17 Renminnan Road 3rd Section, Chengdu, 61041 China; 3 Township Health Center of Shanquan Town Longquanyi District, Chengdu, 610100 China; 4 Health Promotion and Food Nutrition & Safety Key Laboratory of Sichuan Province, Sichuan University, Chengdu, 610041 China; 5 Children’s Medicine Key Laboratory of Sichuan Province, Sichuan University, Chengdu, 610041 China

**Keywords:** Gestational diabetes mellitus, Type 2 diabetes, GWAS-by-subtraction, Genome-wide association study, Genomic structural equation model

## Abstract

**Background:**

The etiology of gestational diabetes mellitus (GDM) is constituted by both type 2 diabetes (T2D)-dependent and T2D-independent mechanisms. However, existing studies have evaluated the impact of T2D-dependent loci only for GDM, which limits the power to assess to what extent genetic variants or biological pathways are specific to GDM.

**Methods:**

We subtracted the genetic effects of a genome-wide association study (GWAS) data for T2D from a GWAS data for GDM to reveal loci linked with T2D-independent components of GDM using genomic structural equation model (SEM). In the discovery stage of GWAS-by-subtraction, we used GWAS summary statistics of GDM and T2D from the FinnGen study as input latent variables (16,802 GDM cases and 237,816 controls; 71,728 T2D cases and 369,007 controls). In the replication stage, we adopted the summary statistics from a previously reported GWAS for GDM and T2D as input (21,263 GDM cases and 301,918 controls; 50,409 T2D cases and 523,897 controls). We functionally annotated variants with genome-wide significance, and performed comprehensive analyses including transcriptome-wide and proteome-wide associations, summary-data-based mendelian randomization, linkage disequilibrium score regression, and Mendelian randomization, to explore genetic patterns for T2D-dependent and -independent components of GDM.

**Results:**

We found 69 independent genome-wide significant loci associated with the T2D-independent components of GDM for discovery stage, of which 49 SNPs are not significant in the GWAS for GDM used as input. T2D-independent components of GDM showed only genetic correlation with birth weight dependent on maternal genetic effect (rg = 0.19; *P* = 0.01), whilst T2D-dependent components of GDM showed genetic correlation with birth weight dependent on fetal genetic effect (rg = -0.22; *P* = 4.76 × 10^− 7^). Loci of T2D-independent components of GDM effects map to genes related to islets of Langerhans, neural stem cells, blood levels of mannose, abundance of *Streptococcus thermophilus* and *Bacteroides vulgatus*, glucose and carbohydrate homeostasis, and the MAPK cascade and adenylate cyclase-activating G protein-coupled receptor signaling pathway.

**Conclusions:**

We identified independent genetic components, loci and genes of GDM that may have been previously masked by T2D, providing more accurate targets for early detection and management of GDM.

**Supplementary Information:**

The online version contains supplementary material available at 10.1186/s13073-026-01674-2.

## Background

Gestational diabetes mellitus (GDM) is a prevalent disorder with a significant rise across various populations over the past 15 years [1]. GDM shows strong genetic correlation with type 2 diabetes (T2D), and is related to an increased risk of T2D, with about a third of females developing T2D within 15 years of their GDM diagnosis [2]. While GDM and T2D in part share a polygenic predisposition, there is a second category of GDM genetic risk factors that are predominantly gestational contributors. The largest existing genome-wide association study (GWAS) of GDM revealed that the genetics of GDM falls into two categories: conventional T2D genetic etiology and etiology predominantly influenced by pregnancy [3]. Yet, existing studies only evaluated the impact of T2D loci, and lack of consistent and reliable measurements of T2D-independent components of GDM in existing genetic datasets pose challenge to control glycemic disturbed by natural perturbation of pregnancy.

**Fig. 1:**
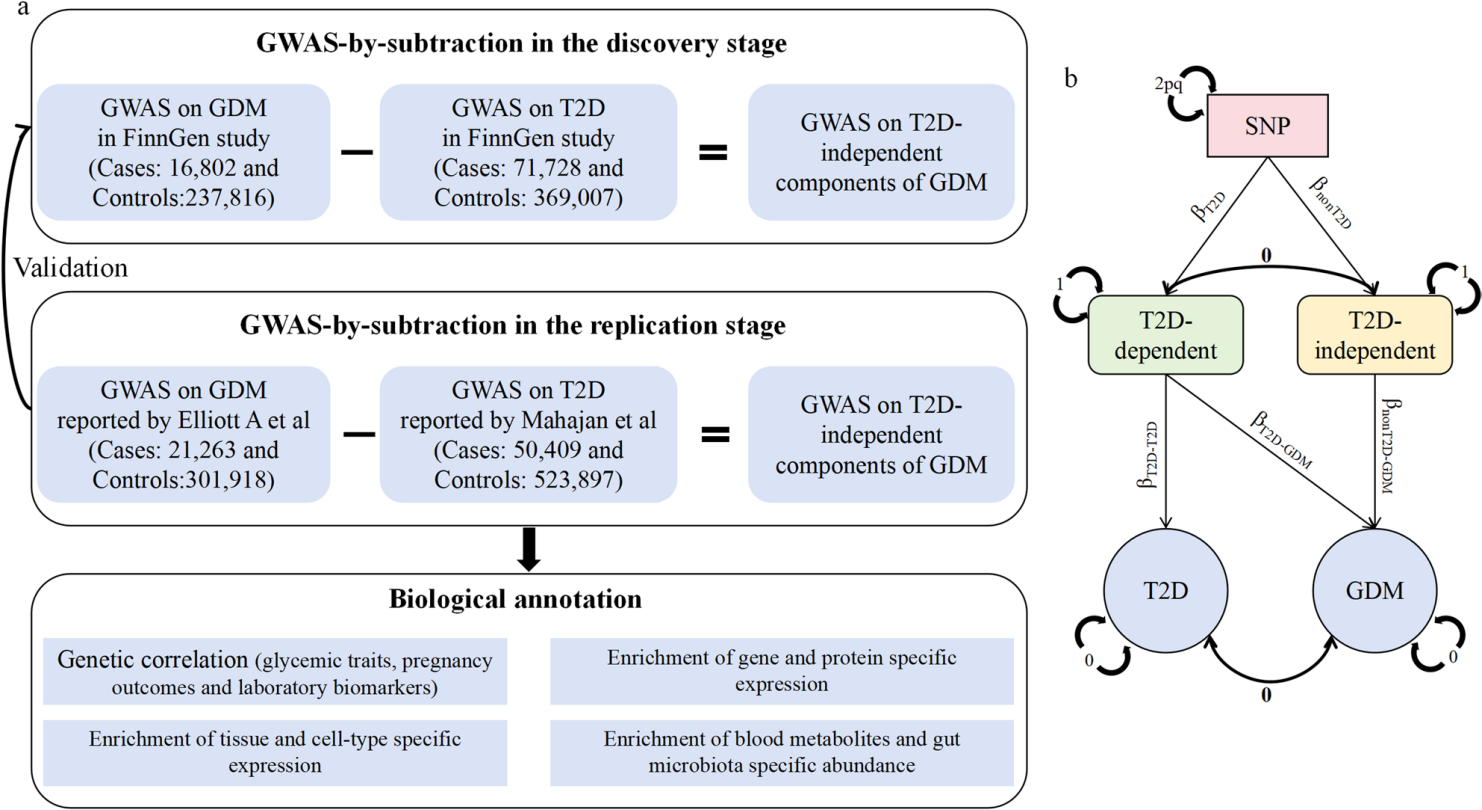
Overall study design. **a** Flowchart of the analysis process. **b** Cholesky model as fitted in Genomic SEM, with path estimates for a single SNP included as illustration. SNP, T2D and GDM are observed variables based on GWAS summary statistics. The genetic covariance between T2D and GDM is estimated based on GWAS summary statistics for T2D and GDM. The model is fitted to a 3 × 3 observed variance-covariance matrix (i.e. SNP, T2D and GDM). T2D dependent and T2D independent are latent (unobserved) variables. The covariances between T2D dependent and between T2D dependent and T2D independent are fixed to 0. The variance of the SNP is fixed to the value of 2pq (p = reference allele frequency, q = alternative allele frequency, based on 1000 Genomes phase 3). The residual variances of T2D and GDM are fixed to 0, so that all variance is explained by the latent factors. The variances of the latent factors are fixed to 1. T2D, type 2 diabetes; GDM, gestational diabetes mellitus

Although recent GWAS for GDM identified 13 loci with GDM-predominant effects using heterogeneity test, 11 loci were previously well-established T2D related loci [3]. Therefore, GWAS of GDM cases and healthy controls may not be the most effective way to delineate the genetic architecture of the non-conventional T2D-independent components of GDM. GWAS-by-subtraction, a genomic structural equation model (SEM), can facilitate a GWAS of a latent trait, i.e. a sub-level trait not measured in any of the genotyped subjects [4], and has been used to comprehend non-intraocular pressure-dependent of primary open angle glaucoma and non-cognitive skills influenced educational success [4, 5]. Hence, we determined the genetic impacts of T2D-independent components, and considered these effects as the genetic variations in GDM not previously explained by T2D (Fig. 1a).

## Methods

### GWAS-by-subtraction

The objective of our GWAS-by-subtraction analysis, conducted by genomic SEM [4, 6], was to estimate, for each SNP, the association with GDM that was independent of that SNP’s association with T2D (hereafter, the T2D-independent SNP effect). The model regressed the GDM and T2D GWAS summary statistics on two latent variables, T2D-dependent and T2D-independent, which were then regressed on each SNP in the genome. This analysis allowed for two paths of association with GDM for each SNP. One path was fully mediated by T2D. The other path was independent of T2D and measured the T2D-independent SNP effect. The principle of our GWAS-by-subtraction is based on previous theoretical basis [4] (Fig. 1b). And recently cross-trait genetic architectures analysis for GDM and T2D suggest no evidence of a sex-specific classification for T2D [3]. Therefore, we used T2D GWAS including female and male in the GWAS-by-subtraction to increase more comprehensive SNP effect for T2D. Table 1 shows information for contributing GWAS summary statistics.

**Table 1:** A list of Contributing Genome-Wide Association Studies

Phenotype	Ancestry	Used in	Soured	Cases	Control
GDM	European	Discovery GWAS for GWAS-by-subtraction	FinnGen study [7]	16,802	237,816
T2D	European	Discovery GWAS for GWAS-by-subtraction	FinnGen study [7]	71,728	369,007
GDM	European	Replication GWAS for GWAS-by-subtraction	Elliott A et al. [3]	21,263	301,918
T2D	European	Replication GWAS for GWAS-by-subtraction	Mahajan A et al.[8]	50,409	523,897
GDM	East Asian	Replication GWAS for GWAS-by-subtraction	Gu Y et al. [9]	12,024	67,845
T2D	East Asian	Replication GWAS for GWAS-by-subtraction	Spracklen CN et al. [10]	54,481	224,231

*T2D* Type 2 diabetes, *GDM* Gestational diabetes mellitus

In the discovery stage of GWAS-by-subtraction, we used GWAS summary statistics of GDM and T2D from the FinnGen study as input latent variables (16,802 GDM cases and 237,816 controls; 71,728 T2D cases and 369,007 controls) [7]. In brief, FinnGen individuals have been genotyped with Illumina and Affymetrix chip arrays [7]. Quality control was performed to remove samples and variants of poor quality. Imputation was performed using a population-specific SISu v3 imputation reference panel [7]. FinnGen GWAS was performed using regenie [7]. Sex, age, 10 principal components, and genotyping batch were included as covariates in the analysis. In the replication stage of GWAS-by-subtraction, we used GWAS summary statistics of GDM reported by Elliott A et al. (21,263 cases and 301,918 controls) and T2D reported by Mahajan et al. (50,409 cases and 523,897 controls) as input latent variables [3, 8]. GWAS GDM included individuals from FinnGen and Estonian Biobank. Genotyping and GWAS for FinnGen individuals are in line with the discovery stage of GWAS-by-subtraction. All Estonian Biobank participants have been genotyped at the core genotyping lab of the institute of genomics, university of Tartu, using illumina global screening array [3]. Samples were genotyped, and plink format files were created using illumina genomestudio [3]. Association analysis was carried out using scalable and accurate implementation of generalized mixed model, using sex, age, and ten principal components as covariates. GWAS T2D were generated using additive logistic regression under an additive genetic model, conducted separately within each of 32 contributing studies of European ancestry [8]. Statistical models were adjusted for body mass index, age, sex, and study-specific genetic principal components to account for population stratification. There was no sample overlap between the discovery and replication GWAS for T2D.

To further validated our findings are in Asian populations, we used Asian populations GWAS summary statistics of GDM reported by Gu Y et al. (12,024 cases and 67,845), and T2D reported by Spracklen CN et al. (54,481 cases and 224,231 controls) as input latent variables in GWAS-by-subtraction analysis [9, 10]. In brief, GWAS for GDM in Asian individuals were generated using low-depth non-invasive prenatal testing sequencing data. Genotypes were imputed with genotype likelihood-based method for phasing and imputation using sparse estimation using a 10,000-sample Chinese reference panel [9]. Primary association testing used plink2 logistic or linear regression under an additive model, adjusted for maternal age, BMI, gestational week, and top 10 principal components and regenie and bayesian optimal linear mixed model were used for validation. GWAS for T2D in Asian individuals using an additive genetic model within a logistic regression framework. Models adjusted for body mass index, age, sex, study-specific covariates, and principal components to account for population stratification.

GWAS-by-subtraction analysis was performed in three stages. At stage 1, we prepared two univariate input GWASs and used multivariate extension of cross-trait linkage disequilibrium (LD) score regression to generate the empirical genetic covariance matrix between the two traits as inputs for the GWAS-by-subtraction model. We restricted four univariate input summary statistics to the HapMap3 variants for estimating genetic covariance and the sampling covariance matrix in LD score regression with a minor allele frequency (MAF) > 0.01 to remove rare SNPs. At stage 2, we removed SNPs with a MAF < 0.01 (to avoid error due to fewer samples within the genotype cluster), SNPs with effect estimates exactly equal to zero, SNPs not matching the 1000 Genomes Phase 3 European or East Asian reference panel, and SNPs with mismatched alleles. Subsequently, we performed genomic SEM GWAS-by-subtraction analysis, in which SNPs were simultaneously regressed on the T2D-dependent and T2D-independent latent variables. The genetic correlation (rg) between the T2D-dependent and T2D-independent latent factors was constrained to zero. In sensitivity analyses, rg was set as 0.1 or 0.2 to explore how robust the results were to this parameter.

From the output GWAS summary statistics, SNPs with *P* < 5 × 10^− 8^ were considered as genome-wide significant. We used *P* < 1 × 10^−^³ as a nominal threshold for directional consistency between discovery and replication, not for genome-wide significance. The lead SNP in each region was identified by clumping the genome-wide significant SNPs using a threshold r^2^ < 0.1 for pairs of SNPs located within 250 kb using functional mapping and annotation (FUMA) [11]. The cross-trait effects of the T2D dependent and T2D independent were further investigated in the GWAS catalog by retrieving all association results for SNPs in high LD (r^2^ ≥ 0.8; European ancestry) with the leading SNPs and were genome-wide significant at *P* < 5.0 × 10^− 8^.

### Genetic correlation

SNP heritability (the phenotype variance explained by a specific set of GWAS variants) and genetic correlations were estimated by LD score regression [12, 13]. To explore genetic difference for glycemic traits with T2D-dependent and T2D-independent components of GDM, we collected the GWAS summary statistics from the Meta-Analyses of Glucose and Insulin-related traits Consortium, including 2-h glucose after an oral glucose challenge [14], fasting glucose [14], fasting insulin [14], glycated hemoglobin [14], fasting proinsulin [15], indices of β-cell function [16], insulin resistance [16], insulin fold change [17], insulin sensitivity index [17], proinsulin [18] and random glucose [19]. To explore genetic difference for pregnancy outcomes with T2D-dependent and T2D-independent components of GDM, we collected the GWAS summary statistics from the Early Growth Genetics Consortium, including birth length [20], preterm delivery [21], gestational duration [21], post-term delivery [21], head circumference [22], fetal birth weight [23], fetal effect birth weight [23], maternal birth weight [23], maternal effect birth weight [23], the last gestational weight [24], fetal effect placental weight [25], and maternal effect placental weight [25]. To explore the network of laboratory biomarkers to which T2D-independent components of GDM was genetically correlated, and which diverged from genetic correlations with T2D-dependent components of GDM, we collected the GWAS summary statistics from a previous study [26]. GWAS summary statistic for GDM, or the summary statistic for the T2D-dependent or T2D-independent components of GDM generated by GWAS-by-subtraction was treated as input summary statistics. Genetic correlations between the GDM traits and a series of traits were calculated.

### Collecting, processing and statistical analysis of clinical profiles

We collected clinical profiles from the information system of West China Second Hospital, Sichuan University. We extracted information on mother and their newborns who were in their first pregnancy and had a live birth, excluded those with unclear age, height, weight, and diagnostic information, leading to 2,881 profiles (374 with pre-pregnancy T2D and 1081 with GDM). We found that the data did not satisfy normality or chi-square, so the Kruskal-Wallis test was chosen to compare the between-group variability, and Dunn’s tests were used to make multiple group comparisons.

### Biological annotation

The goal of biological annotation of GWAS generated by discovery stage is to elucidate molecular mechanisms mediating genetic influences on the phenotype of interest. Our biological annotation analysis proceeded in three steps. First, we conducted enrichment analysis to test if some tissues and cell-types were more likely to mediate T2D-dependent and T2D-independent components of GDM heritability. Second, we conducted transcriptome wide association study (TWAS) [27], proteome wide association study (PWAS) [28] and summary-data-based mendelian randomization (SMR) [29] analysis to explore how the T2D-dependent and T2D-independent components of GDM genetics related to different genes transcription and proteins expression. Third, we conducted metabolome wide association study (MWAS) [30] and mendelian randomization (MR) [31] analysis to explore how the T2D-dependent and T2D-independent components of GDM related to blood metabolites and gut microbiota.

### Enrichment of tissue and cell-type specific expression

To confirm the role of specific tissues or cell**s** in mediating the impacts of T2D-dependent and T2D-independent components on GDM, we used previously defined gene-sets to test for the enrichment of genes specifically expressed in one of 53 genotype-tissue expression (GTEx) tissues or cell types and 152 Franke tissues or cell types captured by aggregation of RNA-seq studies [32]. The tissue-specific and cell-type specific analysis for T2D-dependent and T2D-independent components of GDM was conducted by LD score regression [13, 32].

### Enrichment of gene and protein specific expression

To elaborate the role of specific genes and pathways in mediating the influences of T2D-dependent and T2D-independent components on GDM, firstly, we annotated the closet gene to the lead SNP using annotation of variants annotations, and mapped genes to functional pathways using the Metascape [11, 33]. To further expand our scope for gene discovery, we performed a TWAS and PWAS for T2D-dependent and T2D-independent components of GDM using Functional Summary-based Imputation (FUSION) [27]. The FUSION method involves three steps: (1) identify gene or protein expression features that are cis-heritable (i.e., variants associated with gene expression or protein within or near the genomic locus); (2) construct a linear predictor for each cis-heritable gene or protein (i.e., a SNP-based prediction weight of the gene or protein feature); and (3) calculate both TWAS test statistics incorporating these SNP-based prediction weights and summary-level GWAS Z scores. We used pre-computed blood gene and protein expression weights available, generated from the GTEx (version 8) and the atherosclerosis risk in communities study [27, 28], respectively, and performed bonferroni correction.

We then considered a more conservative approach, SMR, to prioritize specific genes and proteins that could be causally linked to T2D-dependent and T2D-independent components of GDM through leveraging summary-level SNP data of blood from multi-omics studies [29]. We used pre-computed expression quantitative trait loci (QTL) from and protein abundance QTL, generated from the PsychENCODE cohort and fenland cohort [34], respectively, and performed bonferroni correction based on the number of genes tested.

### Enrichment of blood metabolites and gut microbiota specific abundance

To explain the role of metabolites abundance in mediating the effects of T2D-dependent and T2D-independent components on GDM, we performed a MWAS for T2D-dependent and T2D-independent components of GDM using FUSION [27, 30]. We used available pre-computed blood metabolites prediction weights, generated the TwinsUK dataset [31], and performed bonferroni correction. Meanwhile, given the compositions of gut microbiota are influenced by GDM, we used MR to explore the role of specific composition of gut microbiota in mediating the influences of T2D-dependent and T2D-independent components on GDM. We collected available GWAS for the composition of gut bacterial and gut bacterial pathway abundance [35]. We implemented MR analyses via TwoSampleMR (v.0.6.2) packages [36]. For MR analysis, we utilized the inverse-variance weighted (IVW) as main method to estimate the effect of T2D-dependent and T2D-independent components of GDM on gut microbiota abundance [37]. We used weighted median [38], weighted mode [39], and the MR-Egger methods [40] as sensitivity analysis.

## Results

### T2D-independent components for GDM

**Fig. 2:**
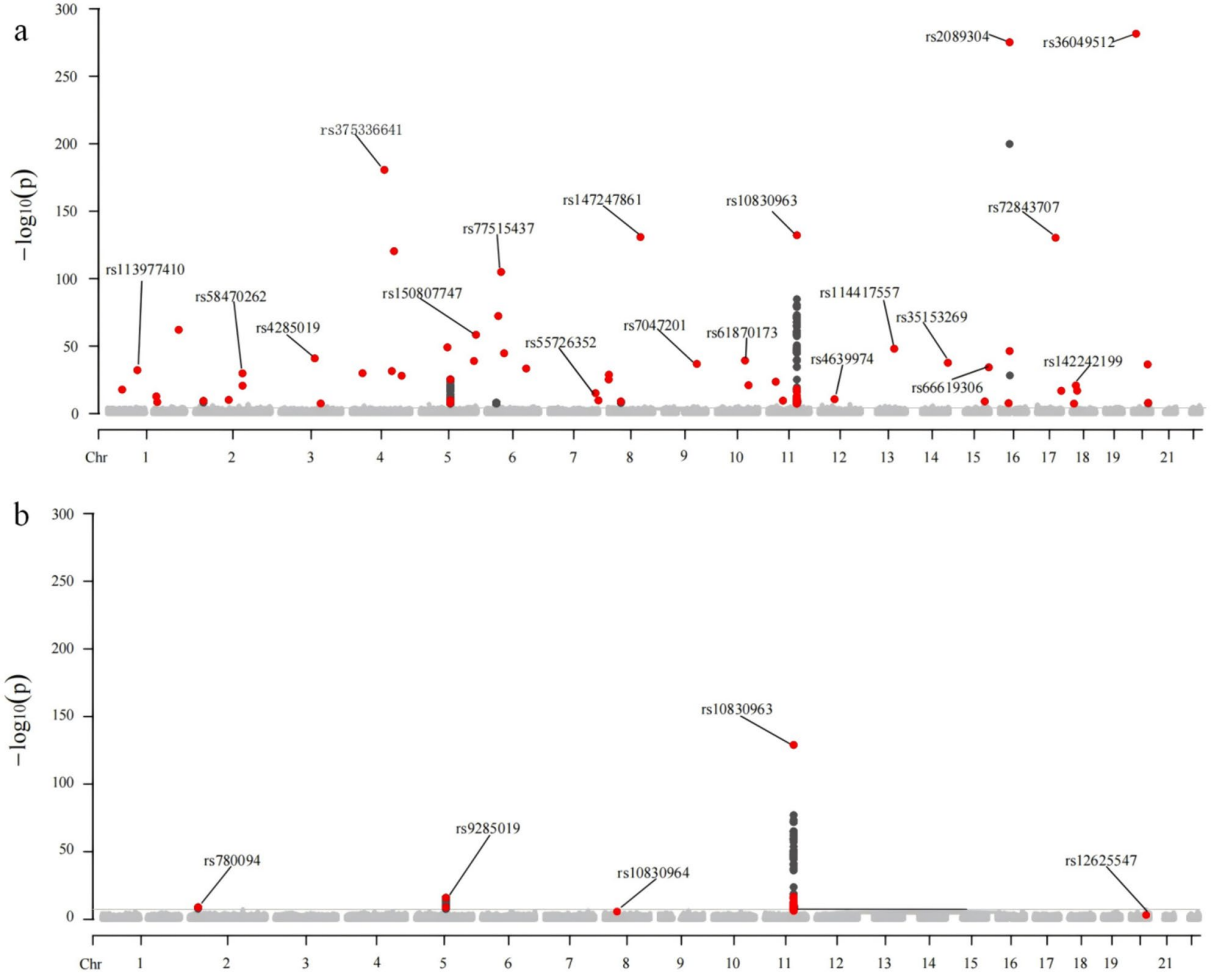
Manhattan plot for the GWAS-by-subtraction derived T2D-independent component of GDM. **a** Manhattan plot for the GWAS-by-subtraction derived T2D-independent component of GDM in the discovery stage. **b** Manhattan plot for the GWAS-by-subtraction derived T2D-independent component of GDM in the replication stage. Plot of the -log10(P-value) associated with Wald’s test (two-sided) of the beta coefficient for the T2D-independent component of GDM for all SNPs, ordered by chromosome and base position. The threshold for genome-wide significance after considering of multiple tests is indicated by the grey dashed line (*P* = 5 × 10^− 8^). Red dots represent lead SNPs

In the discovery stage, GWAS-by-subtraction identified 69 lead SNPs (*P* < 5 × 10^− 8^) from 51 separate regions that are associated with the T2D-independent components of GDM (Table 2; Fig. 2a). The SNP heritability for the T2D-independent components of GDM estimated by LDSC is 5.35%, with no evidence of inflation of test statistics due to population structure (LDSC intercept = 1.02). Notably, 49 of 69 lead SNPs are not significant in the GWAS for GDM used as input for the GWAS-by-subtraction analysis (Table 2). This demonstrated that our approach is beyond simply separating known GDM loci into T2D-dependent and T2D-independent categories. In the replication stage, GWAS-by-subtraction analysis identified 42 independent significant SNPs (*P* < 5.0 × 10^− 8^) (Fig. 2b, Additional file 1: Table S1). There are 20 lead SNPs with nominal significance (*P* < 1 × 10^− 3^) for discovery and replication stage (Table 2). An additional 45 SNPs were selected as proxies for those not present for discovery and replication stage (Additional file 1: Table S2), supporting 65 out of 69 discovery signals. In addition, to validated our findings in non-European populations, we performed GWAS-subtraction analysis on GDM and T2D in Asian populations, and found that 6 of 20 lead SNPs, identified in European populations, are associated with the T2D-independent components of GDM (*P* < 1 × 10^− 5^), including rs10830963, rs10830964, rs12270363, rs4526739, rs636696, rs780094, rs9285019 (Table 2). However, only rs12270363 and rs780094 exhibited effect sizes consistent with the direction observed in European populations. The reasons for these may be that there are fundamental differences in the pathophysiological mechanisms and disease phenotypes of T2D between East Asian and European populations [41, 42]. East Asian populations develop the disease at a lower BMI with more significant visceral fat accumulation, and β-cell dysfunction is the core driving factor for its onset, which is markedly distinct from the pathological characteristics of European and American populations. Given that genetic association analyses of GDM and T2D are highly dependent on the pathophysiological basis of the diseases, such ethnic differences in phenotypes and mechanisms render the relevant results of GWAS subtraction analysis non-transferable across racial groups.

**Table 2:** Genome-wide significant lead variants for the non-T2D-dependent component of GDM

SNP	EA	NEA	Locus	CHR	POS	MAF_EUR_	BETA_EUR_——EUR	SE_EUR_	*P*_EUR_	Function	Nearest Gene	*P*_GDM_	*P*_replication_	BETA_EAS_	SE_EAS_	*P*_EAS_
rs10154014	C	T	50	20	48,356,146	0.139	2.143	0.168	4.03 × 10^− 37^	intergenic	*B4GALT5*	0.089	-	-	-	-
rs10830963	C	G	36	11	92,708,710	0.288	-0.692	0.028	6.76 × 10^− 133^	intron	*MTNR1B*	5.87 × 10^− 216^	1.11 × 10^− 129^	0.657	0.039	1.67 × 10^− 63^
rs10830964	C	T	36	11	92,719,681	0.147	0.31	0.037	5.35 × 10^− 17^	downstream	*MTNR1B*	5.38 × 10^− 25^	7.80 × 10^− 12^	-0.324	0.064	3.59 × 10^− 7^
rs10831057	A	C	36	11	93,267,564	0.197	-0.264	0.038	6.53 × 10^− 12^	intron	*SMCO4*	1.35 × 10^− 18^	-	0.006	0.051	9.09 × 10^− 1^
rs11020131	A	G	36	11	92,713,376	0.02	-0.535	0.06	5.73 × 10^− 19^	intron	*MTNR1B*	4.57 × 10^− 28^	1.58 × 10^− 11^	-	-	-
rs11190875	A	G	32	10	102,999,459	0.157	1.807	0.189	1.05 × 10^− 21^	intron	*LBX1*	0.034	-	-	-	-
rs112432204	C	T	36	11	92,708,092	0.027	-0.553	0.074	1.1 × 10^− 13^	intron	*MTNR1B*	1.42 × 10^− 21^	1.64 × 10^− 16^	-	-	-
rs112569024	A	T	25	7	157,944,883	0.153	1.487	0.233	1.84 × 10^− 10^	-	*PTPRN2*	0.324	-	-	-	-
rs113150876	A	G	15	4	160,202,839	0.183	1.798	0.162	9.28 × 10^− 29^	intron	*RAPGEF2*	0.061	-	-	-	-
rs113777162	G	T	20	6	38,690,353	0.28	1.997	0.11	5.1 × 10^− 73^	intron	*D-H8*	0.148	-	-	-	-
rs113977410	C	T	5	1	226,826,772	0.08	2.875	0.172	8.47 × 10^− 63^	intron	*STUM*	0.107	-	-	-	-
rs114417557	A	C	38	13	76,326,228	0.13	2.279	0.155	8.97 × 10^− 49^	intron	*LMO7*	0.300	-	-	-	-
rs11578147	A	G	2	1	95,243,022	0.193	1.85	0.155	6.95 × 10^− 33^	intron	*SLC44A3*	0.471	-	-0.400	0.768	6.02 × 10^− 1^
rs12270363	G	T	36	11	92,751,333	0.475	-0.174	0.028	8.51 × 10^− 10^	regulatory	*MTNR1B*	1.58 × 10^− 14^	2.51 × 10^− 9^	-0.219	0.043	3.75 × 10^− 7^
rs12549902	A	G	28	8	41,509,259	0.43	0.174	0.029	1.3 × 10^− 9^	upstream	*NKX6-3*	0.002	1.92 × 10^− 6^	-0.054	0.030	7.04 × 10^− 2^
rs12625547	G	T	51	20	50,154,647	0.186	-0.215	0.038	1.05 × 10^− 8^	intron	*NFATC2*	3.19 × 10^− 6^	6.37 × 10^− 4^	0.060	0.056	2.84 × 10^− 1^
rs12795282	C	T	33	11	25,443,086	0.219	1.64	0.161	2.72 × 10^− 24^	intergenic	*ANO3*	0.262	-	-	-	-
rs12804291	C	T	36	11	92,705,307	0.091	0.325	0.059	3.83 × 10^− 8^	intron	*MTNR1B*	2.73 × 10^− 10^	2.09 × 10^− 8^	-	-	-
rs13167145	G	T	17	5	95,711,140	0.123	0.194	0.03	1.24 × 10^− 10^	intron	*PCSK1*	7.15 × 10^− 13^	-	-	-	-
rs142242199	A	T	47	18	14,679,099	0.106	2.07	0.217	1.53 × 10^− 21^	intergenic	*ANKRD30B*	0.033	-	-	-	-
rs1447349	C	G	36	11	92,676,440	0.065	-0.322	0.05	1.71 × 10^− 10^	intergenic	*MTNR1B*	1.15 × 10^− 18^	1.19 × 10^− 11^	-	-	-
rs146228963	A	G	36	11	92,737,847	0.077	0.317	0.053	2.51 × 10^− 9^	intergenic	*MTNR1B*	8.56 × 10^− 11^	3.44 × 10^− 7^	-	-	-
rs147247861	C	G	29	8	103,743,138	0.479	2.043	0.084	1.49 × 10^− 131^	-	*AP003356.1*	0.407	-	-	-	-
rs148124765	A	T	40	15	84,657,647	0.101	1.701	0.279	1.08 × 10^− 9^	intron	*GOLGA6L4*	0.331	-	-	-	-
rs150205522	A	G	36	11	93,064,637	0.052	-0.391	0.061	1.63 × 10^− 10^	intron	*DEUP1*	4.38 × 10^− 14^	5.72 × 10^− 7^	-	-	-
rs150807747	A	G	19	5	177,267,016	0.48	1.654	0.102	4.09 × 10^− 59^	intron	*FAM153A*	0.624	-	-	-	-
rs154453	C	T	17	5	95,211,426	0.229	-0.193	0.034	1.29 × 10^− 8^	downstream	*GLRX*	1.04 × 10^− 11^	1.32 × 10^− 9^	-0.150	0.079	5.84 × 10^− 2^
rs17400671	A	G	46	18	8,659,445	0.072	1.912	0.349	4.46 × 10^− 8^	intergenic	*MTCL1*	0.225	-	-	-	-
rs183495841	A	G	45	17	77,969,351	0.377	1.298	0.152	1.43 × 10^− 17^	intron	*TBC1D16*	0.053	-	-	-	-
rs199746255	A	T	7	2	108,085,738	0.151	1.539	0.237	7.84 × 10^− 11^	intron	*RGPD4*	0.108	-	-	-	-
rs2089304	C	T	43	16	32,457,793	0.395	2.43	0.068	6.3 × 10^− 276^	-	*PABPC1P13*	0.023	-	-	-	-
rs2405819	A	T	26	8	2,747,064	0.323	1.478	0.14	5.9 × 10^− 26^	intergenic	*MYOM2*	0.312	-	-	-	-
rs2845871	C	T	35	11	92,032,930	0.317	0.185	0.032	4.99 × 10^− 9^	intergenic	*FAT3*	3.86 × 10^− 9^	4.46 × 10^− 9^	-0.017	0.033	6.12 × 10^− 1^
rs28691250	A	C	13	4	128,947,455	0.096	2.284	0.193	3.51 × 10^− 32^	intron	*LARP1B*	0.121	-	-	-	-
rs35153269	A	G	39	14	103,412,652	0.366	1.596	0.123	2.35 × 10^− 38^	intron	*AMN*	0.059	-	0.035	0.581	9.52 × 10^− 1^
rs36049512	C	T	49	20	11,602,755	0.185	2.954	0.082	2.94 × 10^− 282^	downstream	*BTBD3*	0.030	-	-	-	-
rs367810486	C	T	11	4	35,607,479	0.125	2.122	0.184	1.23 × 10^30^	-	*RNU6-573P*	0.323	-	-	-	-
rs371504276	C	T	10	3	129,184,764	0.052	2.114	0.384	3.57 × 10^0^8	-	*IFT122*	0.038	-	-	-	-
rs375336641	A	G	12	4	105,244,753	0.142	2.997	0.104	2.42 × 10^181^	intron	*CXXC4*	0.086	-	-	-	-
rs376750538	C	G	42	16	29,284,832	0.355	1.005	0.179	2 × 10^− 08^	intron	*NPIPB11*	0.266	-	-0.278	0.425	5.13 × 10^− 1^
rs4285019	C	T	9	3	110,166,875	0.115	2.353	0.174	1.22 × 10^− 41^	intergenic	*NECTIN3*	0.181	-	-	-	-
rs4526739	C	T	36	11	92,665,020	0.358	-0.201	0.029	3.95 × 10^− 12^	intergenic	*MTNR1B*	8.27 × 10^− 20^	1.47 × 10^− 13^	-0.386	0.058	3.94 × 10^− 11^
rs4639974	G	T	37	12	48,851,067	0.215	1.345	0.201	2.41 × 10^− 11^	intergenic	*ANP32D*	0.413	-	-	-	-
rs4863928	A	C	14	4	135,997,446	0.267	2.273	0.097	4.31 × 10^− 121^	intron	*PABPC4L*	0.328	-	-	-	-
rs5020059	A	T	3	1	155,507,544	0.085	2.008	0.273	1.91 × 10^− 13^	intron	*ASH1L*	0.260	-	-	-	-
rs55726352	G	T	24	7	148,740,846	0.294	1.334	0.165	7.52 × 10^− 16^	intergenic	*PDIA4*	0.468	-	-	-	-
rs56043976	C	G	4	1	158,593,909	0.414	1.071	0.181	3.05 × 10^− 09^	intron	*OR10Z1*	0.161	-	-	-	-
rs58470262	C	T	8	2	152,143,842	0.233	1.558	0.164	2.3 × 10^− 21^	-	*NMI*	0.186	-	-	-	-
rs61870173	A	C	31	10	91,823,836	0.104	2.447	0.185	5.14 × 10^− 40^	intergenic	*KIF20B*	0.005	-	0.002	0.074	9.82 × 10^− 1^
rs61919163	A	G	36	11	93,071,705	0.067	-0.302	0.048	3.13 × 10^− 10^	intron	*DEUP1*	5.07 × 10^− 14^	2.78 × 10^− 8^	0.011	0.032	7.22 × 10^− 1^
rs62381642	C	G	18	5	170,799,941	0.133	2.21	0.168	1.11 × 10^− 39^	intergenic	*NPM1*	0.464	-	-	-	-
rs636696	G	T	34	11	48,435,748	0.412	0.545	0.086	2.88 × 10^− 10^	intergenic	*OR4C5*	0.404	-	0.226	0.089	1.13 × 10^− 2^
rs66619306	C	G	41	15	97,521,162	0.3	1.607	0.13	5.92 × 10^− 35^	intergenic	*NR2F2*	0.583	-	-	-	-
rs66715133	C	G	36	11	92,668,132	0.12	0.332	0.051	6.23 × 10^− 11^	upstream	*MTNR1B*	8.21 × 10^− 14^	5.36 × 10^− 8^	-0.032	0.092	7.31 × 10^− 1^
rs6698457	A	T	1	1	46,741,639	0.016	3.463	0.395	1.84 × 10^− 18^	intron	*LRRC41*	0.418	-	-	-	-
rs67907831	A	C	27	8	3,471,259	0.205	1.76	0.156	1.69 × 10^− 29^	intron	*CSMD1*	0.103	-	-	-	-
rs7047201	C	T	30	9	108,307,717	0.103	2.376	0.186	1.62 × 10^− 37^	-	*FSD1L*	0.454	-	-	-	-
rs71208329	C	T	8	2	151,915,828	0.036	3.079	0.268	1.6 × 10^− 30^	intergenic	*RBM43*	0.035	-	0.322	0.409	4.31 × 10^− 1^
rs72843707	A	C	44	17	59,901,157	0.194	2.518	0.103	5.13 × 10^− 13^1	intron	*BRIP1*	0.115	-	-	-	-
rs74584198	C	T	22	6	57,309,137	0.179	2.016	0.143	2.12 × 10^− 45^	-	*PRIM2*	0.643	-	-	-	-
rs75211051	A	T	23	6	127,668,660	0.187	1.891	0.155	4.08 × 10^− 34^	-	*ECHDC1*	0.010	-	-	-	-
rs77055503	A	C	16	5	86,304,226	0.077	2.83	0.191	8.53 × 10^− 50^	intron	*RASA1*	0.363	-	-	-	-
rs77515437	A	C	21	6	47,662,280	0.154	2.515	0.115	1.17 × 10^− 105^	intron	*ADGRF4*	0.302	-	-	-	-
rs77695435	C	G	48	18	18,512,213	0.389	1.28	0.15	1.19 × 10^− 17^	-	*ROCK1*	0.189	-	-	-	-
rs780094	C	T	6	2	27,741,237	0.411	-0.186	0.03	4.43 × 10^− 10^	intron	*C2orf16*	8.26 × 10^− 19^	1.25 × 10^− 9^	-0.142	0.030	2.54 × 10^− 6^
rs78020513	C	T	35	11	92,089,311	0.034	-0.462	0.084	4.07 × 10^− 08^	intron	*FAT3*	4.54 × 10^− 09^	1.39 × 10^− 7^	-	-	-
rs78265553	A	G	36	11	92,732,391	0.031	-0.464	0.068	1.01 × 10^− 11^	intergenic	*MTNR1B*	1.10 × 10^− 18^	1.08 × 10^− 12^	-	-	-
rs80067804	C	T	43	16	32,526,341	0.486	1.617	0.112	4.98 × 10^− 47^	-	*ABCD1P3*	0.421	-	-	-	-
rs9285019	C	T	17	5	95,719,294	0.284	0.324	0.031	5.55 × 10^− 26^	intron	*PCSK1*	4.46 × 10^− 32^	1.24 × 10^− 16^	-0.136	0.029	1.91 × 10^− 6^

*EA* Effect allele, *NEA *Non-effect allele, *CHR* Chromosome, *POS* Position, *MAF* Minor allele frequency, *BETA*_EUR,_ the effect value for GWAS of T2D-independent GDM in European populations, *SE*_EUR,_ the standard error value for GWAS of T2D-independent GDM in European populations, *P*_EUR,_ the *P* value for GWAS of T2D-independent GDM in European populations, *P*_GDM,_ the *P* value for GWAS of original GDM in European populations, Preplication, the *P* value for GWAS of T2D-independent GDM at replication stage in European populations, *BETA*_EAS,_ the effect value for GWAS of T2D-independent GDM in East Asian populations, *SE*_EUR,_ the standard error value for GWAS of T2D-independent GDM in East Asian populations, *P*_EUR,_ the P value for GWAS of T2D-independent GDM in East Asian populations

Sensitivity analyses in which the genetic correlation between the T2D-dependent and T2D-independent latent factors for GDM was less tightly constrained (rg = 0.1 or 0.2) during the GWAS-by-subtraction analysis led to progressively more SNPs being associated with the T2D-independent components of GDM at *P* < 5 × 10^− 8^ (Additional file 1: Table S3-4; Additional file 2: Fig. S1-S2). In addition, there are 279 independent genome-wide significant SNPs from 139 regions associated with the T2D-dependent components of GDM (Additional file 1: Table S5; Additional file 2: Fig. S3). Only 15 of 279 lead SNPs are significant in the GWAS for GDM used as input for the GWAS-by-subtraction analysis. And there are 47 lead loci for GDM in the original study, in which 7 loci were significant in the GWAS for T2D-independent GDM; 13 loci were significant in the GWAS for T2D-dependent GDM. In total, there are 29 GDM loci which were not among their T2D-independent or -dependent loci. There is minimal evidence of inflation due to population stratification (LDSC intercept = 0.99). The QQ plots for results of GWAS-by-subtraction analysis showed at Additional file 2: Fig. S4. The correlation between the initial GWAS for GDM and the GWAS-by-subtraction results for the T2D-dependent components of GDM is high (rg = 0.76, *P* < 0.001). The SNP heritability of GDM was estimated to be 13.0%. Using GWAS-by-subtraction, the SNP heritability of the T2D-dependent component of GDM was 13.5%, and that of the T2D-independent component was 5.35%. The overall GDM heritability reflects the total additive genetic variance of the GDM phenotype, whereas the component heritabilities represent the genetic variances of two orthogonal latent factors constructed using GWAS-by-subtraction. They are not simple additive partitions of the total GDM heritability, and direct numerical comparison between the overall GDM heritability and the component heritability is not appropriate.

### Association of T2D-dependent and -independent components of GDM with other traits

Seven significant SNPs from four regions for the T2D-independent components of GDM directly are associated to blood glucose or diabetes-related traits in previous GWAS analyses, and 18 significant SNPs from six regions demonstrate pleiotropic effects on other traits (Additional file 1: Table S6). For example, the lead SNP at the *MTNR1B* locus, rs10830956, is in strong LD with a known T2D-associated variant, rs7112766, suggesting that *MTNR1B* or a nearby gene may influence susceptibility to GDM through pleiotropic mechanisms that are both T2D dependent and independent. Many of the significant T2D-independent SNPs are associated to blood glucose-related traits, such as fasting blood glucose, glycated hemoglobin levels, and insulin levels. In contrast, many of the significant T2D-dependent SNPs are associated to diabetes-related traits, such as T2D and medication use in diabetes (Additional file 1: Table S7). In addition, many T2D-dependent SNPs are associated to cognitive traits and reproductive behaviors, while T2D-independent SNPs do not demonstrate such associations (Additional file 1: Table S6-7).

**Fig. 3:**
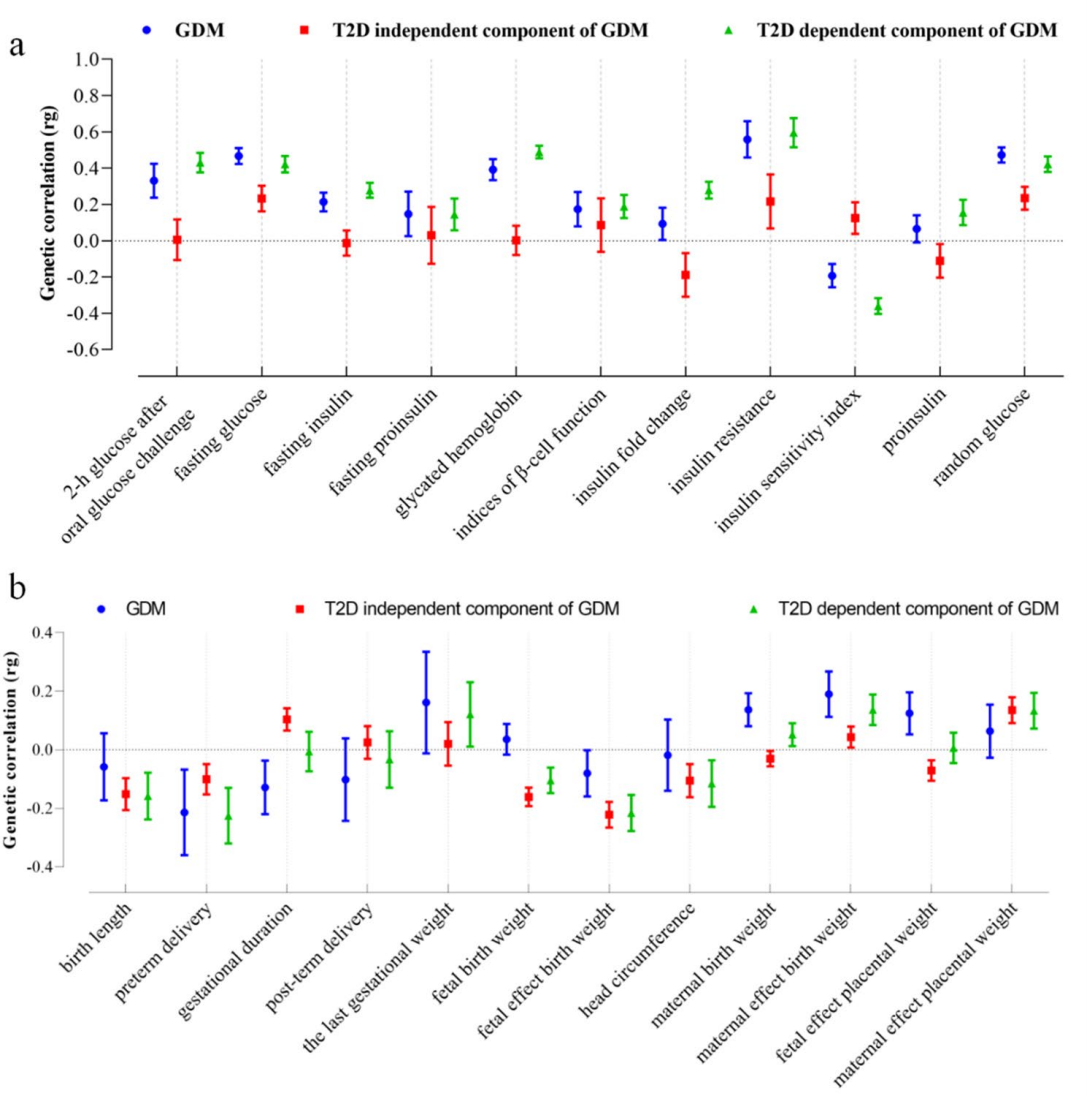
Genetic correlation of the T2D-dependent component of GDM, the T2D-independent component of GDM, and GDM itself with other traits. **a** Genetic correlation of the T2D-dependent component of GDM, the T2D-independent component of GDM, and GDM itself with glycemic traits. **b** Genetic correlation of the T2D-dependent component of GDM, the T2D-independent component of GDM, and GDM itself with pregnancy outcomes. blue is GDM; red is T2D-independent component of GDM; green is T2D-dependent component of GDM. T2D, type 2 diabetes; GDM, gestational diabetes mellitus

The genetic correlation between GDM and various glycemic traits is moderate-to-high: fasting glucose (rg = 0.47; *P* = 2.21 × 10^− 26^), glycated hemoglobin (rg = 0.39; *P* = 1.02 × 10^− 11^), insulin resistance (rg = 0.56; *P* = 2.46 × 10^− 8^), fasting insulin (rg = 0.21; *P* = 2.68 × 10^− 5^), random glucose (rg = 0.47; *P* = 8.71 × 10^− 30^) and 2-h glucose after an oral glucose challenge (rg = 0.33; *P* = 3.52 × 10^− 4^). In agreement with our previous results, genetic correlations between the T2D-dependent components of GDM and these glycemic traits are moderate-to-high: fasting glucose (rg = 0.42; *P* = 4.85 × 10^− 21^), glycated hemoglobin (rg = 0.49; *P* = 6.07 × 10^− 44^), insulin resistance (rg = 0.60; *P* = 6.32 × 10^− 14^), fasting insulin (rg = 0.28; *P* = 1.44 × 10^− 11^), random glucose (rg = 0.42; *P* = 5.03 × 10^− 23^) and 2-h glucose after an oral glucose challenge (rg = 0.43; *P* = 2.32 × 10^− 15^) (Fig. 3a; Additional file 1: Table S8). In contrast, the genetic correlations between the T2D-independent components of GDM and these glycemic traits are notably lower, at about half the magnitude observed for the T2D-dependent components: fasting glucose (rg = 0.23; *P* = 9.00 × 10^− 4^), and random glucose (rg = 0.24; *P* = 1.86 × 10^− 4^) (Fig. 3a; Additional file 1: Table S8).

We found distinct association patterns for the T2D-dependent and T2D-independent components of GDM and pregnancy outcomes (Fig. 3b). We first analyzed clinical profiles of 2,881 Chinese pregnant mother aged 19–48 years. Pregnant mother with GDM showed significant difference at preterm birth rate and offspring birth length compared to those with pre-pregnancy T2D (Additional file 1: Table S9). In genetic correlation analysis, GDM demonstrates a low, while nominal statistically significant, genetic correlation with birth length (rg = -0.16, *P* = 0.047), birth weight dependent on fetal genetic effect (rg = -0.22, *P* = 4.74 × 10^− 4^), birth weight dependent on maternal genetic effect (rg = 0.14, *P* = 9.06 × 10^− 3^), preterm delivery (rg = -0.23, *P* = 0.018), and placental weight (rg = 0.134, *P* = 0.029). T2D-dependent components of GDM demonstrate genetic correlation with birth length (rg = -0.15, *P* = 5.55 × 10^− 3^), birth weight dependent on fetal genetic effect (rg = -0.22, *P* = 4.76 × 10^− 7^), gestational duration (rg = 0.10, *P* = 6.26 × 10^− 3^), and placental weight (rg = 0.14, *P* = 2.24 × 10^− 3^), whilst T2D-independent components of GDM demonstrate genetic correlation with birth weight dependent on maternal genetic effect (rg = 0.19, *P* = 0.014) (Additional file 1: Table S10).

We observed significant genetic correlations for 13 blood laboratory values phenotypically related to GDM (Additional file 1: Table S11; Additional file 2: Fig. S5). Consistent with the results described above, genetic correlations between the T2D-dependent components of GDM and these blood laboratory values were significant (Additional file 1: Table S12; Additional file 2: Fig. S6). In contrast, we only observed genetic correlations between the T2D-independent components of GDM and urea (rg = -0.18, *P* = 2.05 × 10^− 4^) and glucose (rg = 0.26, *P* = 7.33 × 10^− 5^) (Additional file 1: Table S13; Additional file 2: Fig. S7).

### T2D-dependent and -independent components of GDM in tissues and cells

**Fig. 4:**
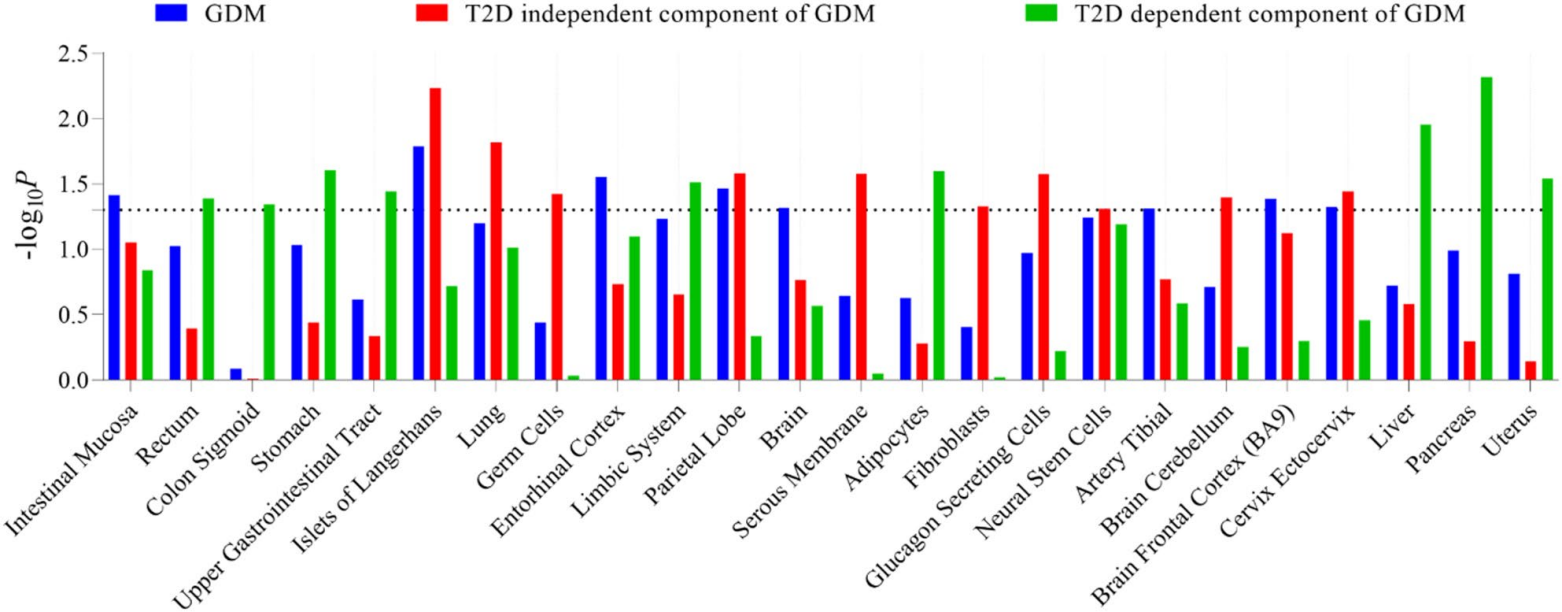
Enrichment of tissue and cell-type specific expression for the T2D-dependent component of GDM, the T2D-independent component of GDM, and GDM itself. The threshold for genome-wide significance is indicated by the grey dashed line. blue is GDM; red is T2D-independent component of GDM; green is T2D-dependent component of GDM. T2D, type 2 diabetes; GDM, gestational diabetes mellitus

We tested whether common variants in genes specifically expressed in 205 tissues and cell types are enriched in the effects on T2D-dependent and T2D-independent components of GDM (Fig. 4; Additional file 1: Table S14-16). GDM demonstrates nominal enrichment in the Langerhans, entorhinal cortex, parietal lobe, intestinal mucosa, brain frontal cortex (BA9), cervix ectocervix, brain and artery tibial. T2D-dependent components of GDM demonstrate nominal enrichment in the pancreas, liver, stomach, adipocytes, uterus, limbic system, upper gastrointestinal tract, rectum and colon sigmoid, whilst T2D-independent components of GDM demonstrate nominal enrichment in the islets of Langerhans, lung, parietal lobe, serous membrane, glucagon secreting cells, cervix ectocervix, germ cells, brain cerebellum, fibroblasts and neural stem cells.

### Genes and proteins of T2D-dependent and -independent components of GDM

There are 60 closest genes annotated to the lead SNPs for T2D-independent components of GDM and 218 closest genes annotated to the lead SNPs for T2D-dependent components of GDM, where 15 genes are overlapped genes for T2D-dependent components and T2D-independent components of GDM, including *GCKR*, *MTNR1B* and *SMCO4* (Additional file 1: Table S17-18). Function pathways enriched for closest genes of T2D-independent components of GDM are not only in glucose and carbohydrate homeostasis, consistent with T2D-dependent components of GDM, but also in mitogen-activated protein kinase cascade and adenylate cyclase-activating G protein-coupled receptor signaling (Additional file 1: Table S19-20; Additional file 2: Fig. S8-9).

**Fig. 5:**
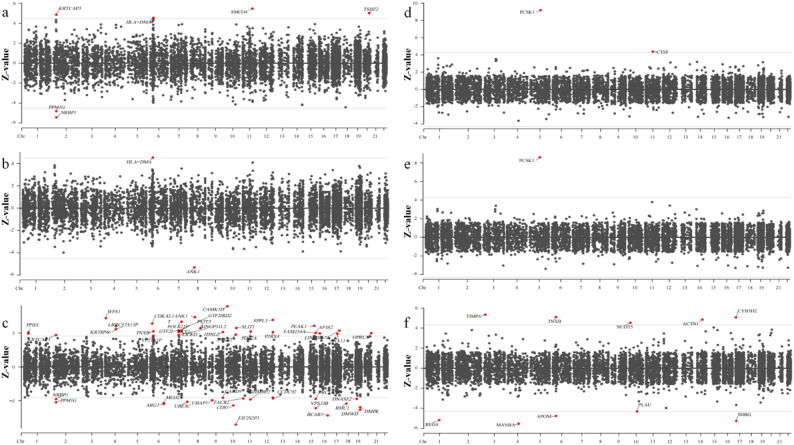
Manhattan plot for the TWAS and PWAS of the T2D-dependent component of GDM, the T2D-independent component of GDM, and GDM itself. **a** Manhattan plot for TWAS for GDM. **b** Manhattan plot for TWAS for T2D-independent component of GDM. **c** Manhattan plot for TWAS for T2D-dependent component of GDM. **d** Manhattan plot for PWAS for GDM. **e** Manhattan plot for PWAS for T2D-independent component of GDM. **f** Manhattan plot for PWAS for T2D-dependent component of GDM. Plot of the Z values associated with Wald’s test (two-sided) of the beta coefficient for all genes or proteins, ordered by chromosome and base position. The threshold for genome-wide significance after considering of multiple tests is indicated by the grey dashed line. Red dots represent significant genes or proteins

We tested whether genetic effects on gene and protein expression in blood, are enriched in the effects on T2D-dependent and T2D-independent components of GDM using TWAS and PWAS. At gene level, GDM demonstrates six genes (*SMCO4*, *NRBP1*, *TSHZ2*, *KRTCAP3*, *PPM1G* and *HLA-DMA*) through TWAS (Fig. 5a; Additional file 1: Table S). We found only two genes (*ANK1* and *HLA-DMA*) through TWAS for T2D-independent components of GDM after multiple testing corrections, whilst we identified 54 genes by TWAS for T2D-dependent components of GDM after multiple testing corrections (Fig. 5b-c; Additional file 1: Table S22-23). Genetically predicted mRNA expression of *ANK1* is a shared gene for the T2D-dependent and T2D-independent components of GDM, with opposite effects on T2D-dependent and T2D-independent components of GDM. At protein level, GDM demonstrates two proteins (PCSK1 and CTSF) through PWAS (Fig. 5d; Additional file 1: Table S24). We found only one protein, PCSK1, through PWAS for T2D-independent components of GDM after multiple testing corrections, whilst we identified ten proteins by PWAS for T2D-dependent components of GDM after multiple testing corrections, and there is no shared protein for T2D-dependent and T2D-independent components of GDM (Fig. 5e-f; Additional file 1: Table S25-26).

In SMR analysis, at gene level, there are two SMR significant genes for T2D-independent components of GDM after applying multiple-testing correction (*ANK1* and *MRPL33*), and only *ANK1* passed HEIDI (*P* > 0.01), while there are 61 SMR significant genes for T2D-dependent components of GDM after applying multiple-testing correction, and 29 gene passed HEIDI (*P* > 0.01; Additional file 1: Table S27-28). Consistent with our TWAS results, genetically predicted mRNA expression of *ANK1* demonstrate the opposite effects on T2D-dependent and T2D-independent components of GDM. At protein level, there two SMR significant proteins for T2D-independent components of GDM (PCSK1 and GCKR), which supports the results for PWAS, while there are ten proteins identified for T2D-dependent components of GDM after multiple testing corrections, and GCKR is shared significant protein for T2D-dependent and T2D-independent components of GDM (Additional file 1: Table S29-30).

### Metabolites of T2D-dependent and -independent components of GDM

**Fig. 6:**
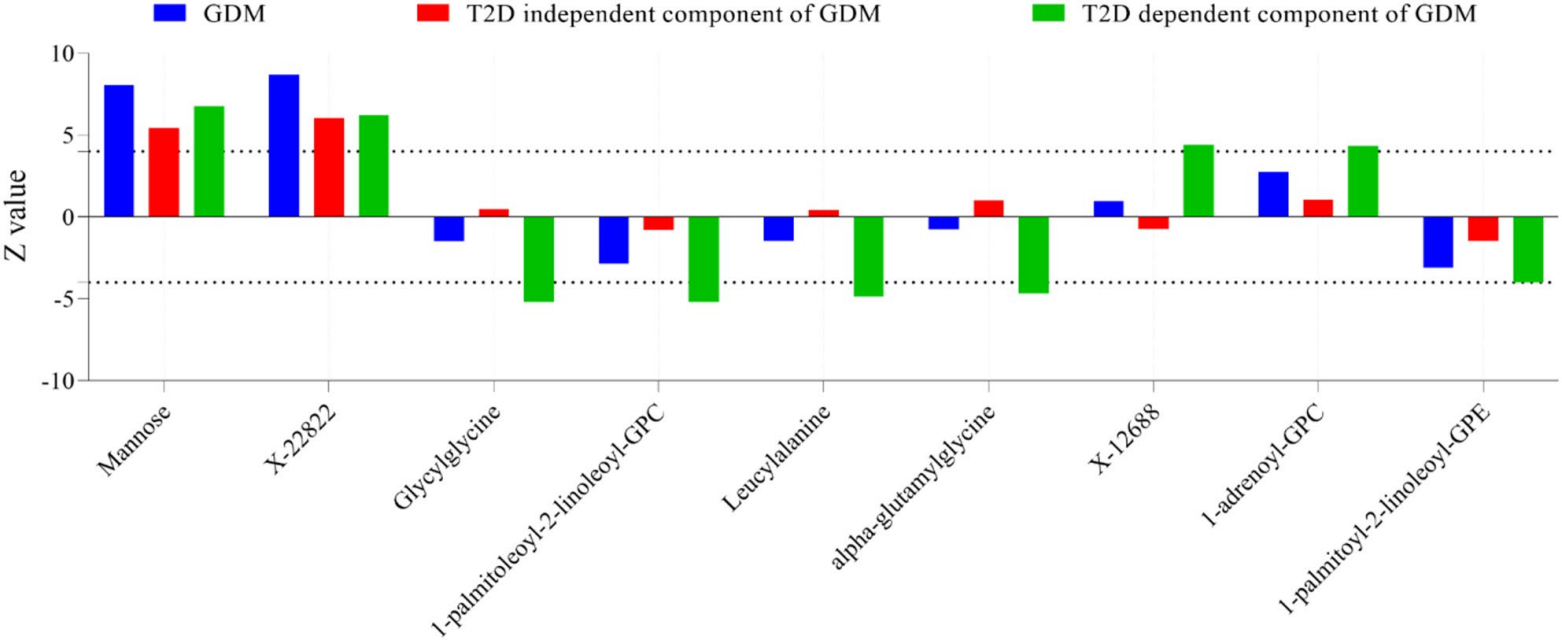
Bar chart of MWAS for the T2D-dependent component of GDM, the T2D-independent component of GDM, and GDM itself. The threshold for significance is indicated by the grey dashed line. blue is GDM; red is T2D-independent component of GDM; green is T2D-dependent component of GDM. T2D, type 2 diabetes; GDM, gestational diabetes mellitus

We tested whether genetic effects on metabolites in blood are enriched in the effects on T2D-dependent and T2D-independent components of GDM using MWAS. GDM demonstrates two metabolites (X-22822 and mannose) through MWAS (Fig. 6; Additional file 1: Table S31), also significant for T2D-independent components of GDM after multiple testing corrections. There are nine metabolites significant for T2D-dependent components of GDM after multiple testing corrections, and X-22,822 and mannose are shared metabolites for T2D-dependent and T2D-independent components of GDM (Fig. 6; Additional file 1: Table S32-33).

### Gut bacterial of T2D-dependent and -independent components of GDM

**Fig. 7:**
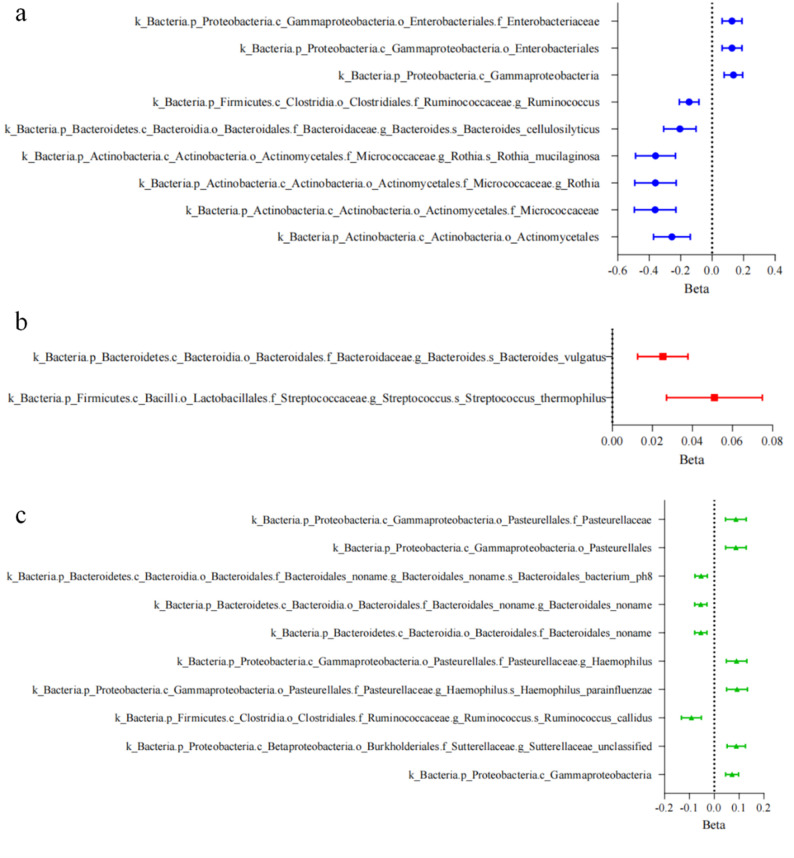
Forest plot of MR for the T2D-dependent component of GDM (**a**), the T2D-independent component of GDM (**b**), and GDM itself (**c**) on gut microbiota abundance. blue is GDM; red is T2D-independent component of GDM; green is T2D-dependent component of GDM. T2D, type 2 diabetes; GDM, gestational diabetes mellitus

We tested if genetic effects on gut bacterial abundance are enriched in the effects on T2D-dependent and T2D-independent components of GDM using MR. GDM demonstrates nominal causal effect on the abundance of nine gut bacterial (Fig. 7a; Additional file 1: Table S34). T2D-independent components of GDM demonstrate nominal causal effect on the abundance of two gut bacterial (*Streptococcus thermophilus* and *Bacteroides vulgatus*). There are ten gut bacterial identified for T2D-dependent components of GDM with nominally significant, and there is no shared gut bacterial for T2D-dependent and T2D-independent components of GDM (Fig. 7b-c; Additional file 1: Table S35-36). For gut bacterial pathway, GDM demonstrates nominal causal effect on the abundance of two gut bacterial pathways. T2D-independent components of GDM demonstrate nominal causal effect on the abundance of five gut bacterial pathways. There are 49 gut bacterial pathways identified for T2D-dependent components of GDM with nominally significant, and pyrimidine deoxyribonucleotides de novo biosynthesis II is a shared gut bacterial pathway for T2D-dependent and T2D-independent components of GDM (Additional file 1: Table S37-39; Additional file 2: Fig. S10-12). In addition, the direction of causal analysis was supported by our sensitivity analysis.

## Discussion

In our study, we employed GWAS-by-subtraction to dissect the genetic risk of GDM into the T2D-independent components and the T2D-dependent components. Unlike single-trait GWAS, GWAS-by-subtraction incorporates multiple traits in a single model, accounting for intricate interrelationships, thereby increasing the probability of uncovering associations specific to underlying mechanisms. We hypothesized that both the T2D-independent and T2D-dependent biological processes contribute to the development of GDM by isolating the T2D-dependent effects on GDM, the residual genetic effects represent distinct components of GDM that is not influenced by T2D. GWAS summary statistics for the T2D-independent component revealed 69 independent SNPs located in 51 distinct genomic regions, which exhibit genome-wide significantly association with GDM through mechanisms presumably unrelated to T2D. The GWAS summary statistics for T2D-dependent components yielded 279 independent variants from 139 unique regions associated with GDM, likely through mechanisms linked to T2D.

A wide range of glycemic traits have been discovered related to GDM, including fasting glucose, hemoglobin A1c and 2-h glucose results on oral glucose tolerance testing [3]. In our study, T2D-dependent components of GDM display similar patterns of association with these glycemic traits, while T2D-independent components of GDM are notably less associated with these glycemic traits, which means glycemic changes in GDM may be more attributable to T2D from genetic perspectives. In addition, T2D-dependent and T2D-independent components of GDM display different patterns of association with pregnancy outcomes. For instance, while T2D-independent components of GDM are predominantly associated to birth weight dependent on maternal genetic effect, rather than with birth weight dependent on fetal genetic effect, the T2D-dependent components share a greater genetic architecture with preterm delivery and placental weight. These findings further supported the idea that GDM may influence offspring growth and development through maternal genetic effects. Shared blood biomarker signatures between GDM and T2D-dependent components further underscore their mechanistic overlap, highlighting maternal genetic effects as a driver of GDM-associated offspring development.

Four novel GDM-associated variants (rs71208329, rs28691250, rs11190875, rs114417557) demonstrate divergent effects: while reducing T2D-dependent GDM risk, they paradoxically increase T2D-independent GDM risk. The T2D-associated variants rs71208329, rs28691250 and rs11190875 would be expected a priori to be associated with a decreased risk of GDM via their effect on T2D. However, our GWAS-by-subtraction analysis revealed that this is not the case and, instead, suggested that these variants are associated with an increased risk of GDM through a T2D-independent mechanism. Our work suggests facilitating effects of these loci; a decreased risk of GDM via an association with lower T2D balanced by an increased risk of GDM via a T2D independent pathway. rs71208329 is located within the *RBM43* gene, which is expressed selectively in white adipose depots that have low thermogenic potential, and is induced by inflammatory cytokines [43, 44]. Adipocyte-selective *RBM43* disruption increases *PGC-1α* translation, resulting in mitochondrial biogenesis and adipose thermogenesis, thus improving glucose homeostasis in mice [45]. rs28691250 is located within the *LARP1B* gene, as part of the protein complex regulating the 5’-TOP mRNA translation, which have higher expression in the β-cells from diabetic patients due to the high metabolic demand during the development of diabetes [46–48]. Nearest gene for rs11190875 is *LBX1*, required for the development of GABAergic interneurons in the dorsal horn of the spinal cord and migration and further development of hypaxial muscle precursor cells for limb muscles, diaphragm and hypoglossal cord [49]. *LBX1* functions as a negative regulator of energy metabolism and overexpression of *LBX1* decreases glucose uptake [50]. Therefore, these evidences suggest that mutations in rs71208329, rs28691250 and rs11190875 may affect glucose homeostasis by affecting the expression of nearest genes, which in turn increases the risk of developing GDM.

Genetic analyses revealed that while T2D-dependent and T2D-independent GDM components are genetically uncorrelated, both traits demonstrate enrichment in pancreas-specific gene sets. However, T2D-independent GDM variants show preferential enrichment in brain-expressed genes, while T2D-dependent variants are enriched in gastrointestinal tract-expressed genes, suggesting distinct neural versus metabolic regulatory mechanisms. At gene level, *ANK1* expression, component of the ankyrin-1 complex involving in the stability and shape of the erythrocyte membrane [51], exhibits opposing effects—negatively associated with T2D-independent GDM but positively associated with the T2D-dependent component-indicating pathway-specific regulatory roles. Variants in the *ANK1* gene result in alterations in the structure of the encoded anchor protein, thereby reducing its plasticity and stability on the erythrocyte membrane, which accelerates the dissolution and destruction of erythrocytes [52]. Associations of *ANK1* variants and T2D were identified by GWAS studies [53–55]. For example, GWAS for Chinese patients found increasing *ANK1* expression in skeletal muscle contributes to T2D susceptibility through regulating energy and lipid homeostasis or skeletal muscle differentiation [54]. However, to date, the role of *ANK1* variants in GDM has not been adequately investigated. Only one study found that *ANK1* expression is the cores down-regulated genes in the placenta of mothers with GDM exchange, thus regulating the occurrence and development of GDM through maintaining cytoskeleton function [56]. *ANK1* expression may play different roles in T2D and GDM through multi-channel and multi-link regulation.

Functionally, T2D-independent GDM genes are enriched not only in glucose homeostasis but also in MAPK cascade and adenylate cyclase-activating G protein-coupled receptor signaling pathways. Inactivating MAPK cascade ameliorates insulin resistance and placental injury in GDM [57], and G protein-coupled receptors (e.g. *GPR1* activating protein kinase B phosphorylation) represent therapeutic targets for GDM [56–61]. Metabolically, elevated mannose levels are a shared risk factor for both GDM components. Elevating mannose level, a bioactive monosaccharide, may be a promising GDM biomarker and intervention target [62]. Notably, elevated mannose levels strongly indicate future risk for various chronic diseases, such as polycystic ovarian syndrome, cardiovascular disease, and albuminuria, potentially contributing to their development rather than serving solely as a novel biomarker [63–65]. Although mechanistic studies on mannose and GDM are currently scarce, circulating mannose levels are positively associated with obesity-independent insulin resistance due to mannose’s interference with insulin receptor function or its role in glycation end-product [66], thereby further increasing GDM risk. In addition, another potential pathway is that mannose could impact the gut microbiome [67]. Moreover, we further investigated gut bacterial abundance effects on T2D-dependent and T2D-independent components of GDM. T2D-independent components of GDM demonstrate nominal causal effect on the abundance of *streptococcus thermophilus* and *bacteroides vulgatus*, which is not identified for GDM. The mannose is a sugar available to *streptococcus thermophilus* which is transported and utilized by *streptococcus thermophilus* through a specific glucose/mannose phosphotransferase system [68], which suggests association between increased abundance of *streptococcus thermophilus* and higher risk of GDM may be attributed to the higher circulating mannose levels.

We acknowledge several limitations. Firstly, GDM GWAS encompasses a relatively limited number of cases, resulting in constrained statistical precision when estimating the correlations between SNPs and GDM risk. Secondly, inclusion of abnormal blood glucose test results from the pregnancy registry in GDM GWAS may lead to discrepancies in SNP heritability. While GWAS-by-subtraction is theoretically anticipated to be resilient to such variations in heritability, we cannot exclude this potential source of inaccuracy. Thirdly, we utilized GWAS summary statistics as input for GWAS-by-subtraction analysis, while it would be more ideal to investigate the causal variants for each trait, given the possibility of subtle differences in tagging causal variants across two GWAS samples. Additionally, GWAS-by-subtraction posits a simplified framework of causal relationships, encompassing only two latent risk factors for GDM: one entirely dependent on T2D and the other fully independent. These relationships are likely far more intricate with potential pleiotropic effects, like common risk factor (body mass index) for both GDM and T2D. Lastly, differences in LD structure and allele frequency between Finnish and other European populations may contribute to lower replication rates for some discovery signals. Future studies should use Finnish-specific LD references and larger Finnish replication data.

## Conclusions

Our study unraveled the genetic risk of GDM into T2D-dependent and T2D-independent components, underlying further insight into GDM mechanism. We identified and explored loci and genes that may have been previously been masked by the effects of T2D, providing more accurate targets for GDM early detection and management.

## Supplementary Information

Additional file 1: Supplementary Tables 1-39 in Excel format.

Additional file 2: Supplementary Figures S1-S12 in PDF format.

## Data Availability

All analyses were based on publicly available data. The GWAS summary statistics produced by GWAS-by-subtraction are available from Zenodo (10.5281/zenodo.19532939). GWAS for GDM and T2D are available from the FinnGen study (https://www.finngen.fi/en/access_results). GWAS for glycemic traits are available from MAGIC (https://magicinvestigators.org/downloads/). GWAS for pregnancy outcomes from the Early Growth Genetics Consortium (http://egg-consortium.org/). Previous GWAS for T2D are available from the DIAGRAM Consortium (https://diagram-consortium.org/downloads.html). GWAS for biomarkers are available form Sinnott-Armstrong et al. on FigShare (https://nih.figshare.com/articles/dataset/The_meta-analyzed_GWAS_summary_statistics_for_35_lab_biomarkers_described_in_Genetics_of_35_blood_and_urine_biomarkers_in_the_UK_Biobank_/12355382). GTEx weights for FUSION analyses are available at https://gusevlab.org/projects/fusion/. Plasma protein weights for FUSION analyses are available at http://nilanjanchatterjeelab.org/pwas/. Metabolites prediction models for FUSION analyses are available at https://github.com/Arthur1021/Metabolites-prediction-models. GWAS for gut bacterial abundance and pathway are available at NHGRI-EBI GWAS Catalog under the study accession numbers GCST90027446-GCST90027857.

## Abbreviations

GDM: Gestational diabetes mellitus
T2D: Type 2 diabetes
GWAS: Genome-wide association study
SEM: Structural equation model
LD: Linkage disequilibrium
FUMA: Functional mapping and annotation
rg: Genetic correlation
MAF: Minor allele frequency
TWAS: Transcriptome wide association study
PWAS: Proteome wide association study
SMR: Summary-data-based mendelian randomization
MWAS: Metabolome wide association study
MR: Mendelian randomization
GTEx: Genotype-tissue expression
FUSION: Functional Summary-based Imputation
QTL: Quantitative trait loci
IVW: Inverse-variance weighted

## References

[1] Shah NS, Wang MC, Freaney PM, Perak AM, Carnethon MR, Kandula NR, et al. Trends in Gestational Diabetes at First Live Birth by Race and Ethnicity in the US, 2011–2019. JAMA. 2021;326(7):660–9. 10.1001/jama.2021.7217. 34402831 10.1001/jama.2021.7217 PMC8371572

[2] Pervjakova N, Moen GH, Borges MC, Ferreira T, Cook JP, Allard C, et al. Multi-ancestry genome-wide association study of gestational diabetes mellitus highlights genetic links with type 2 diabetes. Hum Mol Genet. 2022;31(19):3377–91. 10.1093/hmg/ddac050. 35220425 10.1093/hmg/ddac050 PMC9523562

[3] Elliott A, Walters RK, Pirinen M, Kurki M, Junna N, Goldstein JI, et al. Distinct and shared genetic architectures of gestational diabetes mellitus and type 2 diabetes. Nat Genet. 2024;56(3):377–82. 10.1038/s41588-023-01607-4. 38182742 10.1038/s41588-023-01607-4 PMC10937370

[4] Demange PA, Malanchini M, Mallard TT, Biroli P, Cox SR, Grotzinger AD, et al. Investigating the genetic architecture of noncognitive skills using GWAS-by-subtraction. Nat Genet. 2021;53(1):35–44. 10.1038/s41588-020-00754-2. 33414549 10.1038/s41588-020-00754-2 PMC7116735

[5] Huang Y, Plotnikov D, Wang H, Shi D, Li C, Zhang X, et al. GWAS-by-subtraction reveals an IOP-independent component of primary open angle glaucoma. Nat Commun. 2024;15(1):8962. 10.1038/s41467-024-53331-0. 39419966 10.1038/s41467-024-53331-0 PMC11487129

[6] Grotzinger AD, Rhemtulla M, de Vlaming R, Ritchie SJ, Mallard TT, Hill WD, et al. Genomic structural equation modelling provides insights into the multivariate genetic architecture of complex traits. Nat Hum Behav. 2019;3(5):513–25. 10.1038/s41562-019-0566-x. 30962613 10.1038/s41562-019-0566-x PMC6520146

[7] Kurki MI, Karjalainen J, Palta P, Sipilä TP, Kristiansson K, Donner KM, et al. FinnGen provides genetic insights from a well-phenotyped isolated population. Nature. 2023;613(7944):508–18. 10.1038/s41586-022-05473-8. 36653562 10.1038/s41586-022-05473-8 PMC9849126

[8] Mahajan A, Taliun D, Thurner M, Robertson NR, Torres JM, Rayner NW, et al. Fine-mapping type 2 diabetes loci to single-variant resolution using high-density imputation and islet-specific epigenome maps. Nat Genet. 2018;50(11):1505–13. 10.1038/s41588-018-0241-6. 30297969 10.1038/s41588-018-0241-6 PMC6287706

[9] Gu Y, Zheng H, Wang P, et al. Genetic architecture and risk prediction of gestational diabetes mellitus in Chinese pregnancies. Nat Commun. 2025;16(1):4178. 10.1038/s41467-025-59442-6. 40325049 10.1038/s41467-025-59442-6 PMC12053562

[10] Spracklen CN, Horikoshi M, Kim YJ, et al. Identification of type 2 diabetes loci in 433,540 East Asian individuals. Nature. 2020;582(7811):240–5. 10.1038/s41586-020-2263-3. 32499647 10.1038/s41586-020-2263-3 PMC7292783

[11] Watanabe K, Taskesen E, van Bochoven A, Posthuma D. Functional mapping and annotation of genetic associations with FUMA. Nat Commun. 2017;8(1):1826. 10.1038/s41467-017-01261-5. 29184056 10.1038/s41467-017-01261-5 PMC5705698

[12] Bulik-Sullivan B, Finucane HK, Anttila V, Gusev A, Day FR, Loh PR, et al. An atlas of genetic correlations across human diseases and traits. Nat Genet. 2015;47(11):1236–41. 10.1038/ng.3406. 26414676 10.1038/ng.3406 PMC4797329

[13] Bulik-Sullivan BK, Loh PR, Finucane HK, Ripke S, Yang J, Schizophrenia Working Group of the Psychiatric Genomics Consortium. LD Score regression distinguishes confounding from polygenicity in genome-wide association studies. Nat Genet. 2015;47(3):291–5. 10.1038/ng.3211. 25642630 10.1038/ng.3211 PMC4495769

[14] Chen J, Spracklen CN, Marenne G, et al. The trans-ancestral genomic architecture of glycemic traits. Nat Genet. 2021;53(6):840–60. 10.1038/s41588-021-00852-9. 34059833 10.1038/s41588-021-00852-9 PMC7610958

[15] Strawbridge RJ, Dupuis J, Prokopenko I, et al. Genome-wide association identifies nine common variants associated with fasting proinsulin levels and provides new insights into the pathophysiology of type 2 diabetes. Diabetes. 2011;60(10):2624–34. 10.2337/db11-0415. 21873549 10.2337/db11-0415 PMC3178302

[16] Dupuis J, Langenberg C, Prokopenko I, et al. New genetic loci implicated in fasting glucose homeostasis and their impact on type 2 diabetes risk. Nat Genet. 2010;42(2):105–16. 10.1038/ng.520. 20081858 10.1038/ng.520 PMC3018764

[17] Williamson A, Norris DM, Yin X, et al. Genome-wide association study and functional characterization identifies candidate genes for insulin-stimulated glucose uptake. Nat Genet. 2023;55(6):973–83. 10.1038/s41588-023-01408-9. 37291194 10.1038/s41588-023-01408-9 PMC7614755

[18] Broadaway KA, Yin X, Williamson A, et al. Loci for insulin processing and secretion provide insight into type 2 diabetes risk. Am J Hum Genet. 2023;110(2):284–99. 10.1016/j.ajhg.2023.01.002. 36693378 10.1016/j.ajhg.2023.01.002 PMC9943750

[19] Lagou V, Jiang L, Ulrich A, et al. GWAS of random glucose in 476,326 individuals provide insights into diabetes pathophysiology, complications and treatment stratification. Nat Genet. 2023;55(9):1448–61. 10.1038/s41588-023-01462-3. 37679419 10.1038/s41588-023-01462-3 PMC10484788

[20] van der Valk RJ, Kreiner-Møller E, Kooijman MN, et al. A novel common variant in DCST2 is associated with length in early life and height in adulthood. Hum Mol Genet. 2015;24(4):1155–68. 10.1093/hmg/ddu510. 25281659 10.1093/hmg/ddu510 PMC4447786

[21] Solé-Navais P, Flatley C, Steinthorsdottir V, et al. Genetic effects on the timing of parturition and links to fetal birth weight. Nat Genet. 2023;55(4):559–67. 10.1038/s41588-023-01343-9. 37012456 10.1038/s41588-023-01343-9 PMC10101852

[22] Vogelezang S, Bradfield JP, Early Growth Genetics Consortium, Grant SFA, Felix JF, Jaddoe VWV. Genetics of early-life head circumference and genetic correlations with neurological, psychiatric and cognitive outcomes. BMC Med Genomics. 2022;15(1):124. 10.1186/s12920-022-01281-1. 35659227 10.1186/s12920-022-01281-1 PMC9166310

[23] Warrington NM, Beaumont RN, Horikoshi M, et al. Maternal and fetal genetic effects on birth weight and their relevance to cardio-metabolic risk factors. Nat Genet. 2019;51(5):804–14. 10.1038/s41588-019-0403-1. 31043758 10.1038/s41588-019-0403-1 PMC6522365

[24] Warrington NM, Richmond R, Fenstra B, et al. Maternal and fetal genetic contribution to gestational weight gain. Int J Obes (Lond). 2018;42(4):775–84. 10.1038/ijo.2017.248. 28990592 10.1038/ijo.2017.248 PMC5784805

[25] Beaumont RN, Flatley C, Vaudel M, et al. Genome-wide association study of placental weight identifies distinct and shared genetic influences between placental and fetal growth. Nat Genet. 2023;55(11):1807–19. 10.1038/s41588-023-01520-w. 37798380 10.1038/s41588-023-01520-w PMC10632150

[26] Sinnott-Armstrong N, Tanigawa Y, Amar D, Mars N, Benner C, Aguirre M, et al. Genetics of 35 blood and urine biomarkers in the UK Biobank. Nat Genet. 2021;53(2):185–94. 10.1038/s41588-020-00757-z. 33462484 10.1038/s41588-020-00757-z PMC7867639

[27] Gusev A, Ko A, Shi H, Bhatia G, Chung W, Penninx BW, et al. Integrative approaches for large-scale transcriptome-wide association studies. Nat Genet. 2016;48(3):245–52. 10.1038/ng.3506. 26854917 10.1038/ng.3506 PMC4767558

[28] Zhang J, Dutta D, Köttgen A, Tin A, Schlosser P, Grams ME, et al. Plasma proteome analyses in individuals of European and African ancestry identify cis-pQTLs and models for proteome-wide association studies. Nat Genet. 2022;54(5):593–602. 10.1038/s41588-022-01051-w. 35501419 10.1038/s41588-022-01051-w PMC9236177

[29] Zhu Z, Zhang F, Hu H, Bakshi A, Robinson MR, Powell JE, et al. Integration of summary data from GWAS and eQTL studies predicts complex trait gene targets. Nat Genet. 2016;48(5):481–7. 27019110 10.1038/ng.3538

[30] Liu S, Zhong H, Zhu J, Wu L. Identification of blood metabolites associated with risk of Alzheimer’s disease by integrating genomics and metabolomics data. Mol Psychiatry. 2024;29(4):1153–62. 10.1038/s41380-023-02400-9. 38216726 10.1038/s41380-023-02400-9 PMC11176029

[31] Hemani G, Tilling K, Davey Smith G. Orienting the causal relationship between imprecisely measured traits using GWAS summary data. PLoS Genet. 2017;13(11):e1007081. 10.1371/journal.pgen.1007081. 29149188 10.1371/journal.pgen.1007081 PMC5711033

[32] Finucane HK, Reshef YA, Anttila V, Slowikowski K, Gusev A, Byrnes A, et al. Heritability enrichment of specifically expressed genes identifies disease-relevant tissues and cell types. Nat Genet. 2018;50(4):621–9. 10.1038/s41588-018-0081-4. 29632380 10.1038/s41588-018-0081-4 PMC5896795

[33] Zhou Y, Zhou B, Pache L, Chang M, Khodabakhshi AH, Tanaseichuk O, et al. Metascape provides a biologist-oriented resource for the analysis of systems-level datasets. Nat Commun. 2019;10(1):1523. 10.1038/s41467-019-09234-6. 30944313 10.1038/s41467-019-09234-6 PMC6447622

[34] Guo Y, Xu T, Luo J, Jiang Z, Chen W, Chen H, et al. SMR-Portal: an online platform for integrative analysis of GWAS and xQTL data to identify complex trait genes. Nat Methods. 2025;22(2):220–2. 10.1038/s41592-024-02561-7. 39623049 10.1038/s41592-024-02561-7

[35] Lopera-Maya EA, Kurilshikov A, van der Graaf A, Hu S, Andreu-Sánchez S, Chen L, et al. Effect of host genetics on the gut microbiome in 7,738 participants of the Dutch Microbiome Project. Nat Genet. 2022;54(2):143–51. 10.1038/s41588-021-00992-y. 35115690 10.1038/s41588-021-00992-y

[36] Hemani G, Zheng J, Elsworth B, et al. The MR-Base platform supports systematic causal inference across the human phenome. Elife. 2018;7:e34408. 10.7554/eLife.34408. Published 2018 May 30. 29846171 10.7554/eLife.34408 PMC5976434

[37] Burgess S, Butterworth A, Thompson SG. Mendelian randomization analysis with multiple genetic variants using summarized data. Genet Epidemiol. 2013;37(7):658–65. 10.1002/gepi.21758. 24114802 10.1002/gepi.21758 PMC4377079

[38] Bowden J, Davey Smith G, Haycock PC, Burgess S. Consistent Estimation in Mendelian Randomization with Some Invalid Instruments Using a Weighted Median Estimator. Genet Epidemiol. 2016;40(4):304–14. 10.1002/gepi.21965. 27061298 10.1002/gepi.21965 PMC4849733

[39] Hartwig FP, Davey Smith G, Bowden J. Robust inference in summary data Mendelian randomization via the zero modal pleiotropy assumption. Int J Epidemiol. 2017;46(6):1985–98. 29040600 10.1093/ije/dyx102 PMC5837715

[40] Bowden J, Davey Smith G, Burgess S. Mendelian randomization with invalid instruments: effect estimation and bias detection through Egger regression. Int J Epidemiol. 2015;44(2):512–25. 10.1093/ije/dyv080. 26050253 10.1093/ije/dyv080 PMC4469799

[41] Ma RC, Chan JC. Type 2 diabetes in East Asians: similarities and differences with populations in Europe and the United States. Ann N Y Acad Sci. 10.1111/nyas.12098 10.1111/nyas.12098 PMC3708105 23551121

[42] Zhu Y, Sidell MA, Arterburn D, et al. Racial/Ethnic Disparities in the Prevalence of Diabetes and Prediabetes by BMI: Patient Outcomes Research To Advance Learning (PORTAL) Multisite Cohort of Adults in the U.S. Diabetes Care. 2019;42(12):2211–9. 10.2337/dc19-053244. 31537541 10.2337/dc19-0532 PMC6868463

[43] Roh HC, Tsai LTY, Shao M, Tenen D, Shen Y, Kumari M, et al. Warming Induces Significant Reprogramming of Beige, but Not Brown, Adipocyte Cellular Identity. Cell Metab. 2018;27(5):1121–e375. 10.1016/j.cmet.2018.03.005. 29657031 10.1016/j.cmet.2018.03.005 PMC5932137

[44] Gerstberger S, Hafner M, Tuschl T. A census of human RNA-binding proteins. Nat Rev Genet. 2014;15(12):829–45. 10.1038/nrg3813. 25365966 10.1038/nrg3813 PMC11148870

[45] Dumesic PA, Wilensky SE, Bose S, Van Vranken JG, Gygi SP, Spiegelman BM. RBM43 links adipose inflammation and energy expenditure through translational regulation of PGC1α. bioRxiv [Preprint]. 2023:2023.01.06.522985. 10.1101/2023.01.06.522985.

[46] Werneck-de-Castro JP, Peçanha FLM, Silvestre DH, Bernal-Mizrachi E. The RNA-binding protein LARP1 is dispensable for pancreatic β-cell function and mass. Sci Rep. 2021;11(1):2079. 10.1038/s41598-021-81457-4. 33483593 10.1038/s41598-021-81457-4 PMC7822907

[47] Tcherkezian J, Cargnello M, Romeo Y, Huttlin EL, Lavoie G, Gygi SP, et al. Proteomic analysis of cap-dependent translation identifies LARP1 as a key regulator of 5’TOP mRNA translation. Genes Dev. 2014;28(4):357–71. 10.1101/gad.231407.113. 24532714 10.1101/gad.231407.113 PMC3937514

[48] Hong S, Freeberg MA, Han T, Kamath A, Yao Y, Fukuda T, et al. LARP1 functions as a molecular switch for mTORC1-mediated translation of an essential class of mRNAs. Elife. 2017;6:e25237. 10.7554/eLife.25237. 28650797 10.7554/eLife.25237 PMC5484620

[49] Janusz P, Tokłowicz M, Andrusiewicz M, Kotwicka M, Kotwicki T. Association of LBX1 Gene Methylation Level with Disease Severity in Patients with Idiopathic Scoliosis: Study on Deep Paravertebral Muscles. Genes (Basel). 2022;13(9):1556. 10.3390/genes13091556. 36140724 10.3390/genes13091556 PMC9498322

[50] Nakagawa T, Horiuchi K, Kagami K, Kondo S, Isaji M, Matsuhashi Y, et al. The alteration of LBX1 expression is associated with changes in parameters related to energy metabolism in mice. PLoS ONE. 2024;19(8):e0308445. 10.1371/journal.pone.0308445. 39110747 10.1371/journal.pone.0308445 PMC11305531

[51] Vallese F, Kim K, Yen LY, Johnston JD, Noble AJ, Calì T, et al. Architecture of the human erythrocyte ankyrin-1 complex. Nat Struct Mol Biol. 2022;29(7):706–18. 10.1038/s41594-022-00792-w. 35835865 10.1038/s41594-022-00792-w PMC10373098

[52] Xu W, Ma M, Zhao S, Yuan Y, Tian Z. Case of Congenital Hemolytic Anemia with ATP11C and ANK1 Variants. Child (Basel). 2023;10(10):1600. 10.3390/children10101600. 10.3390/children10101600 PMC10605443 37892263

[53] Pierantozzi E, Raucci L, Buonocore S, Rubino EM, Ding Q, Laurino A, et al. Skeletal muscle overexpression of sAnk1.5 in transgenic mice does not predispose to type 2 diabetes. Sci Rep. 2023;13(1):8195. 10.1038/s41598-023-35393-0. 37210436 10.1038/s41598-023-35393-0 PMC10199891

[54] Yan R, Lai S, Yang Y, Shi H, Cai Z, Sorrentino V, et al. A novel type 2 diabetes risk allele increases the promoter activity of the muscle-specific small ankyrin 1 gene. Sci Rep. 2016;6:25105. 10.1038/srep25105. 27121283 10.1038/srep25105 PMC4848520

[55] Imamura M, Maeda S, Yamauchi T, Hara K, Yasuda K, Morizono T, et al. A single-nucleotide polymorphism in ANK1 is associated with susceptibility to type 2 diabetes in Japanese populations. Hum Mol Genet. 2012;21(13):3042–9. 10.1093/hmg/dds113. 22456796 10.1093/hmg/dds113

[56] Ge L, Huang P, Miao H, Yu H, Wu D, Chen F, et al. The new landscape of differentially expression proteins in placenta tissues of gestational diabetes based on iTRAQ proteomics. Placenta. 2023;131:36–48. 10.1016/j.placenta.2022.11.012. 36473392 10.1016/j.placenta.2022.11.012

[57] Zhao A, Yang Y, Yang Y, Chi Z, Sun Y. Circ-ADAM9 Knockdown Reduces Insulin Resistance and Placental Injury in Diabetic Mice via MAPK Pathway Inactivation. Am J Reprod Immunol. 2024;92(5):e70017. 10.1111/aji.70017. 39575501 10.1111/aji.70017

[58] Musa E, Salazar-Petres E, Vatish M, Levitt N, Sferruzzi-Perri AN, Matjila MJ. Kisspeptin signalling and its correlation with placental ultrastructure and clinical outcomes in pregnant South African women with obesity and gestational diabetes. Placenta. 2024;154:49–59. 10.1016/j.placenta.2024.05.138. 38878622 10.1016/j.placenta.2024.05.138

[59] Zhu Y, Huang S, Chai D, Liang L. G protein-coupled receptor 1 participating in the mechanism of mediating gestational diabetes mellitus by phosphorylating the AKT pathway. Open Life Sci. 2024;19(1):20220920. 10.1515/biol-2022-0920. 39220593 10.1515/biol-2022-0920 PMC11365467

[60] He Q, Lin M, Wu Z, Yu R. Predictive value of first-trimester GPR120 levels in gestational diabetes mellitus. Front Endocrinol (Lausanne). 2023;14:1220472. 10.3389/fendo.2023.1220472. 37842292 10.3389/fendo.2023.1220472 PMC10570794

[61] Zhu M, Lv Y, Peng Y, Wu Y, Feng Y, Jia T, et al. GCKR and ADIPOQ gene polymorphisms in women with gestational diabetes mellitus. Acta Diabetol. 2023;60(12):1709–18. 10.1007/s00592-023-02165-1. 37524927 10.1007/s00592-023-02165-1 PMC10587232

[62] Shen HH, Zhang YY, Wang XY, Wang CJ, Wang Y, Ye JF, et al. Potential Causal Association between Plasma Metabolites, Immunophenotypes, and Female Reproductive Disorders: A Two-Sample Mendelian Randomization Analysis. Biomolecules. 2024;14(1):116. 10.3390/biom14010116. 38254716 10.3390/biom14010116 PMC10813709

[63] Feng D, Shi B, Bi F, Sagnelli M, Sun X, Jiao J, et al. Elevated Serum Mannose Levels as a Marker of Polycystic Ovary Syndrome. Front Endocrinol (Lausanne). 2019;10:711. 10.3389/fendo.2019.00711. 31681178 10.3389/fendo.2019.00711 PMC6811522

[64] Mardinoglu A, Stančáková A, Lotta LA, Kuusisto J, Boren J, Blüher M, et al. Plasma Mannose Levels Are Associated with Incident Type 2 Diabetes and Cardiovascular Disease. Cell Metab. 2017;26(2):281–3. 10.1016/j.cmet.2017.07.006. 28768165 10.1016/j.cmet.2017.07.006

[65] Ferrannini E, Marx N, Andreini D, Campi B, Saba A, Gorini M, et al. Mannose as a biomarker of coronary artery disease: Angiographic evidence and clinical significance. Int J Cardiol. 2022;346:86–92. 10.1016/j.ijcard.2021.11.038. 34800594 10.1016/j.ijcard.2021.11.038

[66] Lee S, Zhang C, Kilicarslan M, Piening BD, Bjornson E, Hallström BM, et al. Integrated Network Analysis Reveals an Association between Plasma Mannose Levels and Insulin Resistance. Cell Metab. 2016;24(1):172–84. 10.1016/j.cmet.2016.05.026. 27345421 10.1016/j.cmet.2016.05.026 PMC6666317

[67] Sharma V, Smolin J, Nayak J, Ayala JE, Scott DA, Peterson SN, et al. Mannose Alters Gut Microbiome, Prevents Diet-Induced Obesity, and Improves Host Metabolism. Cell Rep. 2018;24(12):3087–98. 10.1016/j.celrep.2018.08.064. 30231992 10.1016/j.celrep.2018.08.064 PMC6207501

[68] Cochu A, Vadeboncoeur C, Moineau S, Frenette M. Genetic and biochemical characterization of the phosphoenolpyruvate:glucose/mannose phosphotransferase system of Streptococcus thermophilus. Appl Environ Microbiol. 2003;69(9):5423–32. 10.1128/AEM.69.9.5423-5432.2003. 12957931 10.1128/AEM.69.9.5423-5432.2003 PMC194979

